# Effect of quinoa (*Chenopodium quinoa* W.) flour supplementation in breads on the lipid profile and glycemic index: an *in vivo* study

**DOI:** 10.3389/fnut.2024.1341539

**Published:** 2024-03-26

**Authors:** Natasha R. Marak, Pranati Das, Manashi Das Purkayastha, Luna Dutta Baruah

**Affiliations:** ^1^Department of Food Science and Nutrition, College of Community Science, Central Agricultural University, Tura, Meghalaya, India; ^2^Department of Food Science and Nutrition, College of Community Science, Assam Agricultural University, Jorhat, Assam, India; ^3^Department of Horticulture, College of Agriculture, Assam Agricultural University, Jorhat, Assam, India

**Keywords:** multigrain bread, phenolics, flavonoids, antioxidant, quinoa

## Abstract

Quinoa is a gluten-free pseudocereal, with an excellent nutrient profile containing considerable amounts of fiber and minerals and rich in antioxidants such as polyphenols. The purpose of this research was to investigate the effects of quinoa bread on physical, chemical, bioactive components, glycaemic index (GI), and biochemical parameters. Human subjects aged between 20 and 50 years with the absence of morbid factors were fed daily with quinoa bread for 3 months in order to study its pre-and post-treatment effects on blood glucose, glycosylated haemoglobin, and lipid profile. The effort was made to incorporate the maximum amount of quinoa into the bread without compromising the acceptability of the bread. Of the 14 formulations, TQ13, containing 20% quinoa flour with 3% wheat bran, was selected for further analysis. The GI study revealed that quinoa bread peaked at 45 min with a gradual increase after ingestion of the bread and a steady decline thereafter. The observed value for blood glucose levels, before and after supplementation with quinoa-incorporated bread, was 86.96 ± 15.32 mg/dL and 84.25 ± 18.26 mg/dL, respectively. There was a statistically significant (*p* ≤ 0.05) decrease in levels of triglycerides, total cholesterol, low-density lipoprotein (LDL), and very-LDL (VLDL) level before and after supplementation. However, non-significant changes were observed for high-density lipoprotein levels from the pre- and post-treatment with the quinoa-incorporated bread. Quinoa-incorporated bread possessed low GI (42.00 ± 0.83) compared to control (69.20 ± 1.84) and long-term consumption proved to contain functional efficacies in terms of hypolipidemic effect.

## 1 Introduction

The food industry is always in the process of developing new food products according to the demands of the consumer for products with improved quality. This is also a demand of the hour to produce food products with health benefits to counteract increased incidences of non-communicable diseases all over the world (1). Hence, the emphasis is on the incorporation of functional ingredients into new food products for better quality and benefits (2). Functional foods may be regarded as innovative, physiologically active foods, which can provide additional health benefits beyond basic nutrition. Fortification of food products is one of the major techniques used to create functional food products (2).

Formulation of functional foods with the inclusion of grains from the categories of cereals, millets, pseudocereals, legumes, and oilseeds enhances the nutritional qualities of foods. Furthermore, if these grains are unprocessed or minimally processed, the benefits of whole grains are further increased with probable capabilities of disease prevention (3). Consumption of whole-grain products is associated with reduced incidence of diseases, such as cancer (4), cardiovascular disease (5), high blood pressure (6), and diabetes (7).

Currently, composite or multigrain flours are increasingly being used to produce products such as cookies (8–10) breads (11–13), and cakes (14, 15). Pseudocereal grains such as quinoa, buckwheat, and amaranth are rich in a wide range of compounds such as flavonoids, phenolic acids, fatty acids, trace elements, and vitamins with known effects on human health (16). Quinoa, a native plant belonging to the Andean region, is considered gluten-free with an excellent nutrient profile (17). They contain considerable amounts of fiber (3.8 g/100 g) and minerals, such as calcium (1,487 mg kg^−1^ dry wt) and iron (132 mg kg^−1^ dry wt) (18). Quinoa is also rich in antioxidants such as polyphenols (19). Once known to the Incas as the “mother of all grains,” today quinoa is receiving increasing attention because of its high nutritional quality (20). Efforts to improve the quality of baked goods via substitution of cereal grains for quinoa flour have revealed that up to 10% quinoa flour in breads improves nutritional quality without negatively affecting loaf volume. Considering the lower nutritional value of most gluten-free products in the market, further study on the behavior of quinoa proteins and carbohydrates in bread is warranted (21).

## 2 Materials and methods

### 2.1 Formulation of quinoa flour-incorporated multigrain breads

Control bread was formulated to contain a maximum amount of whole wheat flour without compromising sensory quality. Based on preliminary studies (data not shown), it was found that a 60:40 ratio of refined wheat flour to whole wheat flour performed the best for making quality bread, as compared to that of 100% whole wheat flour bread and also without the use of bread improver and/or enzymes. Therefore, this ratio was used as a control (coded as T_0_) for subsequent studies. Thus, a control bread formulation of a 60:40 ratio of refined wheat flour to whole wheat flour was used as the base. All raw materials were sourced from the local market of Jorhat, Assam. A total of fourteen formulations were developed with 5, 10, 15, 20, 25 and 30 % quinoa flour with and without fenugreek flour and wheat bran (Table 1). Fenugreek seeds were added as it is a rich source of soluble dietary fiber; 100 g of seeds provides more than 65% of dietary fiber and contains saponins, hemicelluloses, mucilage, tannins, and pectin, which help to decrease the level of low-density lipoprotein cholesterol (LDL) in blood by decreasing the reabsorption of bile salts in the colon (22).

**Table 1:** Formulation of quinoa flour incorporated multigrain breads.

Formulation	Refined wheat flour (%)	Whole wheat flour (%)	Quinoa flour (%)	Fenugreek seed flour (%)	Wheat bran (%)
T0	60	40	–	–	–
TQ1	60	35	5	–	–
TQ2	60	30	10	–	–
TQ3	60	25	15	–	–
TQ4	55	25	20	–	–
TQ5	50	25	25	–	–
TQ6	45	25	30	–	–
TQ7	57	35	5	3	–
TQ8	57	30	10	3	–
TQ9	57	25	15	3	–
TQ10	57	20	20	3	–
TQ11	50	30	10	10	–
TQ12	50	20	20	10	–
TQ13	52	25	20	–	3
TQ14	47	25	25	–	3

#### 2.1.1 Sensory evaluation of multigrain breads

Sensory evaluation of the developed quinoa breads was performed by 15 trained and semi-trained panel members from the Department of Food Science and Nutrition, College of Community Science, Assam Agricultural University, Jorhat (23). The panellists were asked to score the products for every quality attribute such as color, texture, taste, flavor, appearance and overall acceptability, using a scorecard of a 9-point Hedonic Rating Scale. The bread was completely cooled and then stored for 24 h before sensory evaluation.

### 2.2 Physical properties of multigrain breads

Loaf weight, loaf volume, specific volume, texture profile, and color were studied for the developed quinoa bread sample.

#### 2.2.1 Loaf weight

The bread is simply weighed in an electronic weighing balance to record the loaf weight (23).

#### 2.2.2 Loaf volume of bread

The loaf volume of bread was measured using the rapeseed replacement method (24). Loaf volume (VL) was calculated according to the following formula:

VL (cm^3^) = VC – VR.

#### 2.2.3 Specific volume of bread

Specific volume is an important parameter in bread making and indicates the final gas retention in the bread and affects consumer preference. The specific volume (VS) of bread was measured by using the following expression:

VS (cm^3^/g) = VL/W.

#### 2.2.4 Texture profile analysis

Texture profile analysis (TPA) was carried out using a texture analyser (TA-XT Plus, Stable Micro Systems, United Kingdom) as adopted by the standard method by AACC (24). The sample was removed from its place of storage and placed centrally over the supports just prior to testing (25, 26). The texture analyser was equipped with a 36-mm-radius probe. The first and second compression cycles indicate the force vs. time data during the first and second compression of the product by the instrumental probe. A P0.5R cylindrical probe with 2 mm/s of pre-test and post-test speeds and 45% compression was taken for TPA. TPA is a two-bite test, which includes the first and second compression cycles. The first and second compression cycles indicate the force vs. time data during the first and second compression of the product by the instrumental probe. Three sets of measurements per loaf for replications were recorded.

#### 2.2.5 Color analysis

Color analysis of multigrain breads was done by using a Hunter Lab colorimeter (model SM-3001476 microsensors). The instrument was calibrated with user-supplied black plate calibration standard that was used for zero setting, and white calibration plates were used for white calibration settings. The instrument was placed at three different exposures at different places. Readings were displayed as L*, a*, and b* color parameters according to the CIELAB system of color measurement. The value of a* ranged from −100 (redness) to +100 (greenness), the b* values ranged from −100 (blueness) to +100 (yellowness), while as L* value indicating the measure of lightness, ranged from 0 (indicating black) to 100 (indicating white) (27). The three values are required to completely describe the color of an object.

#### 2.2.6 Proximate analysis

The analysis of moisture, crude fat, crude protein, crude fiber, and ash was carried out as described in AOAC, 2000. The carbohydrate content was calculated by the difference method. The energy value (kcal) of the bread sample was calculated by the method of Gopalan et al. (28). Total dietary fiber was also estimated as described by AOAC (25).

#### 2.2.7 Estimation of minerals

The minerals calcium and iron were determined by using an atomic absorption spectrophotometer according to the method of AACC (24).

### 2.3 Bioactive components of quinoa bread

#### 2.3.1 Determination of total antioxidant capacity

In total, 2 g of dried sample was extracted with 20 mL of methanol (99.5%). The extraction was done twice each for hours in a shaking machine. The supernatant was filtered using Whatman no. 1 filter paper after centrifuging the suspension at 10,000 rpm for 15 min, the filtrate was stored at -20°C till analysis. A 100 μL of an aliquot of sample extract was taken in a test and add 2.9 mL of DPPH solution (0.005 mM solution of 2,2-diphenyl-1-picryl-hydrazyl prepared in 99.5% methanol); after this solution was added, it was vortex mixed vigorously. The test tube was incubated in the dark for half an hour. Discoloration of DPPH was measured against a blank at 517 nm. Methanol was used as blank, and DPPH methanolic extract was used as standard.

#### 2.3.2 Determination of total phenolic content

Total phenolics were determined spectrophotometrically using Folin–Ciocalteu reagent and expressed as gallic acid equivalent/g (mg of GAE/g of the sample) (28). A known aliquot (0.2 mL) of sample extract was taken, and the volume was made up to 1.5 mL with D/W. To the extract, 0.5 mL of Folin–Ciocalteu reagent was added by addition of 10 mL of 7.5% sodium carbonate solution and mixed well by shaking. Incubated at 37°C for 60 min and absorbance was measured at 750 nm in a spectrophotometer, concentration was calculated from a standard curve prepared from different concentrations of gallic acid (5–20 μg) and distilled water as the blank.

#### 2.3.3 Determination of total flavonoid estimation

Total flavonoid content (TPC) was determined by using the method described by Zhishen et al. (29). A known aliquot of the sample was taken, and the volume was made up to 5 mL with distilled water; 0.3 mL of 5 % of NaNO_2_ was added. After 5 min, 0.6 mL of 10 % AlCl_3_ was added and mixed. After 6 min, 2 mL of 1 N NaOH was added and mixed. Then, 2.1 mL of distilled water was added to make the volume up to 10 mL. The absorbance of the resulting pink color was read at 510 nm against a blank (distilled water), and Rutin (50 μg to 200 μg) was taken as standard.

### 2.4 *In vivo* assessment to study the efficacy of multigrain bread

#### 2.4.1 Glycaemic index

For the estimation of glycemic index, the procedure given by Wolever et al. (30) was followed.

Selection of subjects for the intervention was based on age (20–50 years) and in the absence of morbid factors. The subjects were asked to sign a consent form, and the ethical committee recommended the study vide authorization number AAU/CCS/FSN/IEC/241. Each subject was given 100 g of multigrain bread daily for 3 months in order to study pre- and post-treatment effects on blood glucose, glycosylated haemoglobin, and lipid profile.

#### 2.4.2 Estimation of blood glucose

Blood glucose was estimated by a commercial assay kit (Coral Glucose estimating kit). The blood samples were collected in heparinized sterile centrifuge tubes and were centrifuged at 1107 grams-force (3,000 rpm for 20 min). Serum was collected in a microcentrifuge and estimated by auto analyser using a commercial assay kit (Coral Glucose estimation kit using GOD/POD method). The standard laboratory method of blood glucose estimation was done through a spectrophotometer (V-730 UV–Visible Spectrophotometer).

#### 2.4.3 Measurement of glycosylated haemoglobin

The collected whole-blood samples were assayed for the measurement of glycosylated haemoglobin using high-performance liquid chromatography (LC-4000 Series HPLC model, with the use of the Tosoh A1c 2.2 Plus Glycohemoglobin Analyzer method in 2003–2004 and the Tosoh G7 method in 2007–2008, Tosoh Corp).

#### 2.4.4 Lipid profile analysis

Lipid profile analysis was studied for triglycerides, total cholesterol, HDL, LDL, and VLDL by the method described by Wagner et al. (31).

### 2.5 Legal ethical aspects

The Ethical Committee of the University approved the research study after a thorough discussion, and a certificate was issued for the same.

### 2.6 Statistical analyses

The results are expressed as mean ± SD. Data obtained were statistically analysed by using SPSS statistics (Ver. 20) software using one-way analysis of variance (ANOVA), and the significance of the difference between means of tested parameters was carried out using Duncan’s post-hoc test. Differences were considered statistically significant at the *p* ≤ 0.05 level at a 5% level of significance.

## 3 Results

### 3.1 Sensory evaluation

Of the 14 formulations, TQ1, TQ2, TQ3, TQ4, and TQ13 had similar (*p* ≥ 0.05) scores in all sensory parameters and scores were significantly (*p* ≤ 0.05) higher as compared to all other formulations. Although TQ1, TQ2, TQ3, TQ4, and TQ13 had statistically similar scores (*p* ≥ 0.05), TQ13 was expected to have better nutritional profiles as the quinoa incorporation was higher as compared to TQ1, TQ2, and TQ3. Furthermore, although TQ4 and TQ13 both contained 20% quinoa flour, TQ13 was further incorporated with 3% bran (Table 1) and was selected for further studies. The increase in the percentage of incorporation of quinoa and other ingredients led to the decrease in acceptability (Table 2) of the breads formulated from quinoa flour. In some of the formulations, sensory scores were even lower than 5.

**Table 2:** Sensory evaluation of quinoa flour-incorporated multigrain breads.

Sample name	Sensory score					
	Color	Texture	Taste	Flavor	Appearance	Overall acceptability
TQ1	8.3 ± 0.31^d^	8.2 ± 0.36^e^	8.3 ± 0.45^e^	8.2 ± 0.44^e^	8.3 ± 0.49^f^	8.26 ± 0.55^d^
TQ2	8.3 ± 0.76^d^	8.2 ± 0.38^e^	8.3 ± 0.56^e^	8.3 ± 0.32^e^	8.3 ± 0.4^f^	8.28 ± 0.54^d^
TQ3	8.3 ± 0.53^d^	8.2 ± 0.6^e^	8.1 ± 0.53^e^	8.2 ± 0.59^e^	8.2 ± 0.32^f^	8.2 ± 0.48^d^
TQ4	8.3 ± 0.44^d^	8.0 ± 0.46^e^	8.1 ± 0.41^e^	8.0 ± 0.48^e^	8.2 ± 0.59^f^	8.1 ± 0.62^d^
TQ5	7.0 ± 0.38^c^	4.6 ± 0.43^b^	4.5 ± 0.39^b^	4.5 ± 0.42^c^	5.0 ± 0.42^e^	5.1 ± 0.63^c^
TQ6	6.5 ± 0.56^c^	4.0 ± 0.51^a^	4.0 ± 0.54^b^	4.0 ± 0.48^b^	4.0 ± 0.59^a^	4.5 ± 0.54^b^
TQ7	6.3 ± 0.67^b^	5.3 ± 0.66^c^	5.5 ± 0.56^c^	4.8 ± 0.58^c^	6.0 ± 0.43^c^	5.6 ± 0.57^c^
TQ8	6.2 ± 0.39^b^	5.2 ± 0.46^c^	5.4 ± 0.5^c^	4.8 ± 0.51^c^	4.9 ± 0.47^b^	5.3 ± 0.55^c^
TQ9	6.0 ± 0.48^a^	4.6 ± 0.66^a^	4.4 ± 0.74^b^	4.6 ± 0.49^c^	4.8 ± 0.49^b^	4.9 ± 0.48^b^
TQ10	6.0 ± 0.78^a^	4.0 ± 0.55^a^	3.0 ± 0.67^a^	3.0 ± 0.48^a^	4.0 ± 0.41^a^	4.0 ± 0.67^a^
TQ11	<5	<5	<5	<5	<5	<5
TQ12	<5	<5	<5	<5	<5	<5
TQ13	8.3 ± 0.76^d^	7.0 ± 0.67^e^	7.9 ± 0.64^e^	8.0 ± 0.71^e^	8.0 ± 0.58^f^	8.0 ± 0.56^d^
TQ14	7.0 ± 0.67^c^	5.2 ± 0.73^b^	5.8 ± 0.68^c^	5.0 ± 0.24^c^	5.8 ± 0.45^d^	5.8 ± 0.58^c^
CD at 5%	0.329	0.623	0.463	0.558	0.505	0.564

Values are expressed in mean ± SD (standard deviation). Means within columns separated by Duncan’s multiple range tests. Means followed by the same letter in superscript(s) are not significantly different.

### 3.2 Physical characteristics of quinoa bread

#### 3.2.1 Loaf weight, loaf volume, and specific volume

The loaf weight, loaf volume, and specific volume of the quinoa-incorporated multigrain bread were 434.28 ± 0.56 g, 1300.32 ± 0.65 cm^3^, and 2.99 ± 0.60 cm^3^/g, respectively, whereas those for the control, it was 434.28 g ± 0.56, 1486.32 ± 0.64 cm^3^, and 3.42 ± 0.75 cm^3^/g, respectively. Compared to the control bread sample, quinoa bread had a lesser loaf volume and specific volume but their loaf weights were the same. White bread or refined wheat bread is the commonly consumed form of bread around the globe due to its good physical properties such as loaf volume, but it has less value in terms of nutrition (Table 3).

**Table 3:** Physical characteristics of the quinoa multigrain bread.

Bread sample	Loaf weight (g)	Loaf volume (cm^3^)	Specific volume (cm^3^/g)
T_0_	434.28 ± 0.56	1486.32 ± 0.64	3.42 ± 0.75
TQ13	434.28 ± 0.56	1300.32 ± 0.65	2.99 ± 0.60
*t*-value	–	174.87^*^	93.34^*^

Values are expressed in mean ± SD (standard deviation). ^*^Significant at *p* ≤ 0.05.

#### 3.2.2 Texture profile analysis

Commonly considered parameters used to determine bread texture is the study of hardness, cohesiveness, springiness, and chewiness of the bread. The values obtained for quinoa bread were 1.58 ± 1.19 kg (hardness), 0.95 ± 0.43 s (cohesiveness), 0.94 ± 1.45 (springiness), and 1.42 ± 3.22 kg-s (chewiness). The values for control bread were 0.33 ± 0.56 kg, 0.45 ± 1.22 s, 1.00 ± 0.26, and 0.06 ± 1.27 kg-s for hardness, cohesiveness, springiness, and chewiness, respectively. From Table 4, it can be inferred that the control bread possessed superior quality in terms of the texture of the bread.

**Table 4:** Texture profile analysis of the selected multigrain breads.

Bread samples	Hardness (kg)	Cohesiveness (s)	Springiness	Chewiness kg-s
T_0_	0.33 ± 0.56	0.45 ± 1.22	1.00 ± 0.26	0.06 ± 1.27
TQ13	1.58 ± 1.19	0.95 ± 0.43	0.94 ± 1.45	1.42 ± 3.22
*t*-value	165.92*	106.06^*^	100.46^*^	289.91^*^

Values are expressed in mean ± SD (standard deviation).

#### 3.2.3 Color analysis

The L*, a*, and b* values in regard to the crust color are presented in Table 5. The values obtained for L*, a*, and b* for quinoa bread were 56.64 ± 1.24, 3.30 ± 0.18, and 22.11 ± 0.19, respectively, and those for the control 67.50 ± 1.15, 2.09 ± 0.05, and 16.46 ± 0.58, respectively. The values for the quinoa bread are more intense compared to the control sample. The values for the crumb color of the quinoa bread were 53.64 ± 1.54 (L* value), 13.53 ± 0.58 (a* value), and 31.70 ± 0.19 (b* value) as shown in Table 6. In both the crust and crumb color of the quinoa bread, a* and b* color values that indicate redness and yellowness were more than those of control. The color value for L*, indicating lightness or whiteness was found to be lesser than that of the control bread.

**Table 5:** Color characteristics of the crust of the selected multigrain breads.

Bread sample	L*	a*	b*
T_0_	67.50 ± 1.15	2.09 ± 0.05	16.46 ± 0.58
TQ13	56.64 ± 1.24	3.30 ± 0.18	22.11 ± 0.19
*t*-value	1331.30^*^	150.64^*^	847^*^

a* value ranged from −100 (redness) to +100 (greenness); b* values ranged from −100 (blueness) to +100 (yellowness); L* value indicating the measure of lightness ranged from 0 (black) to 100 (white). Values are expressed in mean ± SD (standard deviation).

**Table 6:** Color characteristics of crumbs of the selected multigrain breads.

Bread sample	L*	a*	b*
T_0_	61.21 ± 1.25	11.51 ± 0.13	13.90 ± 0.65
TQ13	53.87 ± 1.54	13.53 ± 0.58	31.70 ± 0.19
*t*-value	898.96^*^	247.40^*^	2671^*^

a* value ranged from −100 (redness) to +100 (greenness); b* values ranged from −100 (blueness) to +100 (yellowness); L* value indicating the measure of lightness, ranged from 0 (black) to 100 (white). Values are expressed in mean ± SD (standard deviation).

### 3.3 Chemical characteristics of the quinoa bread

The proximate analysis involves the determination of moisture, crude protein, crude fat, crude fiber, and ash of quinoa bread sample. The mean values of quinoa bread were 39.05 ± 0.67 g/100 g (moisture), 4.82 ± 0.41 g/100 g (crude fat), 14.28 ± 1.65 g/100 g (crude protein), 2.53 ± 0.55 g/100 g (crude fiber), 1.15 ± 0.88 g/100 g (total ash), 27.86 ± 0.23 g/100 g (carbohydrate), 302 ± 0.49 kcal (energy), 154.89 ± 0.48 mg/100 g (calcium), 8.49 ± 0.12 mg/100 g (iron), 13.75 ± 0.54 g/100 g (total dietary fiber), 10.56 ± 0.74 g/100 g (insoluble dietary fiber), and 3.19 ± 0.46 g/100 g (soluble dietary fiber). From Table 7, it is concluded that the chemical composition of quinoa bread was higher than the control values, which proved that quinoa is rich in many nutrients (Tables 8–10).

**Table 7:** Proximate compositions of the selected multigrain breads.

Bread samples	Moisture (g/100 g)	Crude fat (g/100 g)	Protein (g/100 g)	Crude fiber (%)	Total ash (g/100 g)	Carbohydrate (g/100 g)	Energy (kcal)
T_0_	34.79 ± 0.62	3.21 ± 0.84	12.74 ± 0.50	1.09 ± 0.62	0.98 ± 0.47	25.98 ± 0.88	252.29 ± 0.44
TQ13	39.05 ± 0.67	4.82 ± 0.41	14.28 ± 1.65	2.53 ± 0.55	1.15 ± 0.88	27.86 ± 0.23	302 ± 0.49
*t*-value	788.97^*^	197.18^*^	188.61^*^	137.24^*^	27.5^*^	141.5^*^	47.12^*^

Values are expressed in mean ± SD (standard deviation). ^*^Significant at *p* ≤ 0.05.

**Table 8:** Mineral content of the selected multigrain breads (mg/100 g, on a dry weight basis).

Bread samples	Calcium (mg/100 g)	Iron (mg/100 g)
T_0_	91.95 ± 1.30	9.85 ± 0.46
TQ13	154.89 ± 0.48	8.49 ± 0.12
*t*-value	770^*^	107.67^*^

Values are expressed in mean ± SD (standard deviation). ^*^Significant at *p* ≤ 0.05.

**Table 9:** Dietary fiber content of selected multigrain breads (g/100 g, on a dry weight basis).

Bread samples	Dietary fiber (g/100 g, on a dry weight basis)		
	Total dietary fiber	Insoluble dietary fiber	Soluble dietary fiber
T_0_	12.10 ± 0.45	9.96 ± 0.57	2.14 ± 0.38
TQ13	13.75 ± 0.54	10.56 ± 0.74	3.19 ± 0.45
*t*-value	171.82^*^	71.03^*^	110.62^*^

Values are expressed in mean ± SD (standard deviation). ^*^Significant at *p* ≤ 0.05.

**Table 10:** Bioactive components of the quinoa multigrain breads.

Bread samples	Total antioxidant capacity (scavenging ability %)	Total phenolics (mg GAE/g)	Total flavonoids (mg QE/g)
T_0_ (Control)	25.88 ± 0.37	1.05 ± 0.22	0.11 ± 1.45
TQ13	33.26 ± 1.53	2.31 ± 1.58	0.22 ± 0.46
*t*-value	591.45^*^	144.76^*^	15.5^*^

### 3.4 Bioactive components of quinoa bread

Antioxidant capacity of quinoa bread was measured by the ability of the test sample to scavenge DPPH radicals. Table 11 shows that quinoa bread had a mean antioxidant capacity of 33.26 ± 1.53 %. Phenolic compounds in cereals are found in free, soluble conjugated, and bound forms. The bound form represents the major proportion of phenolic acid in cereals. In this study, the total phenolic content of the quinoa bread was 2.31 ± 1.58 mg GAE/g. Flavonoids are a group of polyphenolic compounds that are widely distributed and possess health-related properties such as anticancer, antioxidant, anti-inflammatory, and antiviral properties, which are based on their antioxidant activity. The total flavonoid content of quinoa multigrain bread was 0.22 ± 0.46 mg QE/g. In all the three findings, the values were higher than those of the control sample.

**Table 11:** Glycaemic index of selected multigrain breads.

Bread samples	Glycaemic index
T_0_	69.20 ± 1.84
TQ13	42.00 ± 0.83
*t*-value	788.97*

Values are expressed in mean ± SD (standard deviation).

### 3.5 *In vivo* assessment to study the efficacy of multigrain breads

The glycaemic index (GI) is a concept that allows the ranking of carbohydrate-rich foods in terms of their potential to raise blood glucose levels. White bread, which served as the control for determination of GI, peaked at 30 min and remained comparatively high over a 120-min period of investigation. Quinoa bread (TQ13) showed a slower peaking and decline. Quinoa bread was found to peak at 45 min, showing a slower, gradual increase after ingestion of the bread and then showed a steady decline. The observed GI of quinoa bread was 42.00 ± 0.83, which is considered as low-GI foods.

The effect of quinoa-incorporated MG bread having low GI (42.00 ± 0.82) on the blood profile was studied in terms of pre- and post-intervention on blood glucose levels, glycosylated haemoglobin, and lipid profile. A single meal of a low-fiber food like white bread may stimulate high postprandial blood glucose response and influence glucose and insulin metabolism. The observed blood glucose levels, as indicated in Table 12, showed that before supplementation with quinoa-incorporated multigrain bread, the value was 86.96 ± 15.32 mg/dL, and after supplementation, it reduced to 84.25 ± 18.26 mg/dL. Though there was a decrease in the blood glucose levels after supplementation, it was non-significant (*p* ≥ 0.05).

**Table 12:** Biochemical parameters (mg/dl) pre- and post-treatment with quinoa-incorporated multigrain bread.

Biochemical parameters	Before intervention (mg/dl)	After intervention (mg/dl)	*p*-value
Blood glucose	86.96 ± 15.32	84.25 ± 15.26	1.25
Glycated haemoglobin	5.46 ± 0.541	5.01 ± 0.677	0.681
Total cholesterol	180.38 ± 36.08	160.43 ± 31.75	0.002^*^
Triglycerides	175.11 ± 59.60	108.09 ± 39	0.005^*^
HDL	53.17 ± 7.64	52.51 ± 7.03	0.112
LDL	94.02 ± 32.75	87.52 ± 26.19	0.005^*^
VLDL	35.33 ± 12.25	21.67 ± 7.80	0.003^*^

Values are expressed in mean ± SD (standard deviation). ^*^Significant at *p* ≤ 0.05. HDL, high-density lipoprotein; LDL, low-density lipoprotein; VLDL, very-low-density lipoprotein.

Glycosylated haemoglobin is a form of haemoglobin (Hb) that is chemically linked to sugar. Most monosaccharides, including galactose and fructose, spontaneously bond with haemoglobin, when present in the bloodstream of humans. The test for glycosylated haemoglobin as presented in Table 12 showed a decrease in values, but the changes observed were non-significant (p ≥ 0.05). The mean value was 5.46 ± 0.541 mg/dL before supplementation, and it lowered slightly to 5.01 ± 0.677 mg/dL after supplementation. The value observed fell within the normal range (4 and 5.6%).

The lipid profile values observed before and after supplementation with quinoa bread are presented in Table 12. The values obtained before supplementation were 180.38 ± 36.08 mg/dL, 175.11 ± 59.60 mg/dL, 53.17 ± 7.64 mg/dL, 94.02 ± 32.75/dl, and 35.33 ± 12.25 for cholesterol, triglyceride, HDL, LDL, and VLDL, respectively, and those after supplementation were 160.43 ± 31.75 mg/dL, 108.09 ± 39 mg/dL, 52.51 ± 7.03 mg/dL, 87.52 ± 26.19 mg/dL, and 21.67 ± 7.80 mg/dL for cholesterol, triglyceride, HDL, LDL, and VLDL, respectively. A statistically significant (*p* ≤ 0.05) decrease in the levels of triglycerides, total cholesterol, LDL and VLDL was observed. However, there was no significant change observed for HDL levels from the pre- and post-treatment with the quinoa-incorporated multigrain bread (TQ13).

## 4 Discussion

Refined wheat flour is not a good source of protein, minerals, and certain bioactive components as compared to whole quinoa flour; therefore, quinoa flour was incorporated into wheat-based bread to improve nutritional and bioactive properties. The addition of quinoa flour and wheat bran in appropriate proportions improved the chemical properties of wheat bread as well as its nutritional status. Wheat flour bread is deficient or poor in many nutrients. Development and study of the physical and chemical properties and bioactive components of quinoa bread have led to many positive effects on health. The addition of quinoa flour and wheat bran to wheat bread resulted in a nutrient-dense bread that with positive response in terms of decreasing the levels of triglycerides, total cholesterol, LDL, and VLDL significantly.

Some studies have suggested that 20% quinoa inclusion in white bread to have the highest acceptability (31) while some other studies suggest 15% of quinoa (32) and 10% (33) as the most acceptable. In this study, the selected formulation TQ13 had an incorporation of 20% quinoa flour as well as 3% wheat bran and was found to be satisfactory from both sensory and nutritional points of view. Increased bran content was assumed to add to the health benefits of the quinoa bread.

The loaf volume of quinoa bread was lower than that of the control bread. The addition of too much fiber was reported to affect the bread quality when it comes to texture, loaf volume, and appearance (34, 35). High levels of fiber dilute gluten and lowers gas retention causing a decrease in loaf volume. This might be the reason for obtaining a lesser loaf volume in the quinoa bread formulated in this study. Several other studies reported less bread volume due to the incorporation of ingredients, such as finger millet (36), barley (37, 38), and composite flour (39). A good loaf volume is obtained if the gas bubbles in the fermented dough expand with minimal rupturing of the gluten network during proofing and baking. The presence of ß-glucans-a fraction of total dietary fiber in high levels reduces the specific volume of the breads (40), which can be compared to the present study where fiber from quinoa and bran might have reduced the volume. The decrease in volume was proportional to the increase in non-cereal flour. The specific volume decreased with increased incorporation of composite flour (41). The specific volume of bread reveals the development of bread dough after baking. The greater the specific volume value, the more inflated and voluminous is the bread dough after baking.

The hardness of quinoa bread, as seen in Table 4, was higher than the control. The increase in hardness might be attributed to the higher water absorption of fiber-rich-incorporated dough. This can be explained by an interaction between water and hydroxyl groups of polysaccharides through hydrogen bonding (42). Higher hardness with increasing bran addition with regard to dough texture is attributed to the thickening of the walls surrounding the air bubbles in the crumb (34, 43). The cohesiveness of quinoa bread was higher than that of the control bread. Higher cohesiveness in composite breads may be due to higher moisture retention compared to control bread (44). Cohesiveness in composite bread may be attributed to the decreased aeration and compact texture (45). The results of the present investigation were similar to the findings of Abdelghafor et al., (46), Nasar-Abbas and Jayasena (47), and Chhavi and Sarita (48), who reported that chewiness increased progressively with an increase in the level of multigrain flour in the composite bread as compared to the control. This may be attributed to the dilution of wheat gluten with an increased proportion of other flours and added to the weakening of the strength of gluten.

The L*, a*, and b* values in regard to the crust color are presented in Table 5. The values obtained for L*, a* and b* for quinoa bread were 56.64 ± 1.24, 3.30 ± 0.18, and 22.11 ± 0.19 and those for the control 67.50 ± 1.15, 2.09 ± 0.05, and 16.46 ± 0.58. The values for the quinoa bread are more intense compared to the control sample. The values for crumb color (Table 6) of the quinoa bread were 53.64 ± 1.54 (L* value), 13.53 ± 0.58 (a* value), and 31.70 ± 0.19 (b* value) as shown in Table 6. In both the crust and crumb color of the quinoa bread, the a* and b* color values, which indicate redness and yellowness, were more than those of control (49–51). The color value for L*, indicating lightness or whiteness, was found to be lesser than that of the control bread. The increasing darkness and redness of composite breads might be due to the high content of protein in the case of quinoa flour, which resulted in the Maillard browning during baking (52).

The moisture content of quinoa bread (39.05 ± 0.67) is reported to contain a higher value than the control (34.79 ± 0.62). The results of the present investigation are well in accordance with those reported by Otegbayo et al. (53), Ngozi (54), Ameh et al. (55), and Rehman et al., (56), who reported higher moisture in wheat bran-incorporated breads and composite breads. The increased moisture content of composite breads may be a consequence of the increased water absorption capacity of dough (57, 58) and also an increase in fiber content (59). In the present study also, the crude fiber content of quinoa multigrain experimental bread was higher as compared to control bread, which could be the reason for the higher moisture levels of experimental breads in comparison with control breads. The results of the present study are in accordance with a study by Sharma et al. (59) and Sanz-Penella et al. (60) who also observed an increase in fat content in composite breads enriched with millets and pseudocereals. Quinoa has a fat content ranging from 5 to 7 %, thus contributing to the high fat content of quinoa-incorporated breads [65]. In the present study, quinoa MG breads had higher levels of crude fat as compared to control bread. Wright et al. (61) and Comai et al. (62) also reported that quinoa had higher total protein content (12.9–16.5%) compared to other grains and pseudocereals. Other studies have also reported a similar increase in protein content in quinoa composite breads (58, 63). The effects of the addition of whole-grain barley flour to wheat flour reported improved levels of β-glucan (64). Higher fiber content may be due to the fiber content of the individual grains and the wheat bran added to the quinoa bread. The carbohydrate content of quinoa bread was higher than that of the control bread. Composite breads are known to contain higher carbohydrate content compared to control bread (65). The results of the present study are also in agreement with studies by Olaoye et al. (66) and Ambreen et al. (67). The energy content of quinoa bread was 302.2 ± 0.49 kcal. Shehry (68) also reported a higher energy content of quinoa-incorporated breads compared to control. This may be due to the high fat content of quinoa. Graf et al. (21) and Lalit (69) also reported higher energy content in quinoa-based composite breads.

Demin et al. (32) reported that quinoa-supplemented bread had a 40% increase in calcium content compared to the control and the reason might be the calcium content of quinoa, which is 126.94 mg/100 g (70). Similar increasing trends were also observed in other studies (71, 72). Kumari (73) reported 8.60 mg/100 g iron for toast bread incorporated with full-fat rice bran (10%) and 9.20 mg/100 g iron for defatted rice bran (10%). The formulated toast bread was found to have significant results over control bread containing 7.90 mg/100 g iron. Young (74) reported bread prepared from rice bran had an iron content of 9.32–20.52 mg. Naikare (75) prepared bread from a 15% sorghum blend with 85% wheat flour, and the iron content was 3.4 mg/100 g more than that of the control.

Alvarez-Jubete et al. (76) documented the influence of amaranth, quinoa, and buckwheat on polyphenol profile and antioxidant capacity and revealed that buckwheat demonstrated the most antioxidant activity. Quinoa is known to contain phenolics as a major group of secondary metabolites (21), which may be the reason for high values in TQ13. The high TPC of whole grain and bran are due to the presence of pericarp and aleurone layers, which are rich in antioxidant compounds (59). Quinoa-incorporated breads contain higher flavonoids with possible nutraceutical benefits (68). Flavonoids are a group of polyphenolic compounds possessing health-related properties based on their antioxidant activity. Pandey et al. (77) also pointed out the protective role of flavonoids, specifically flavones and flavonols, from cardiovascular and cancer diseases.

As per the classification given by Augustin et al., (78), foods having less than 55 GI are considered as low-GI foods, 56–69 medium-GI foods, and above 70 high-GI foods. Based on the classification, quinoa-incorporated bread (42.00 ± 0.83) can be categorized under low-GI foods. Quinoa, known to contain good amounts of dietary fiber, modulates postprandial insulin response, promotes endogenous cholesterol conversion to bile acids and improves intestinal microbiota. Epidemiological studies have shown an inverse relationship between dietary fiber intake and the development of cardiovascular disease, obesity, and type 2 diabetes (79). The effects of various commercial whole-grain breads on postprandial blood glucose response and GI in healthy subjects reported that whole-grain oat bread exhibited the lowest peaking of blood glucose level and also reported the lowest GI (80). They stated that the oat bread was especially rich not only in total fat and protein but also in dietary fiber compared to other breads under study, which could be the reason for low blood glucose peak and GI. The management of the postprandial blood glucose response is crucial, as high blood glucose response may instigate the incidence of diabetes, obesity, coronary heart diseases, and some types of cancer (81). The observed blood glucose levels, as indicated in Table 12, which shows that before supplementation with quinoa-incorporated multigrain bread, the value was 86.96 ± 15.32 mg/dL, and after supplementation, it reduced to 84.25 ± 18.26 mg/dL. Although there was a decrease in the blood glucose levels after supplementation, it was not significant (*p* ≥ 0.05). This indicates that the usual trend of lowering blood glucose levels with the intervention of high-fiber supplements (82) was observed but not significant (p ≥ 0.05) in the present study. It could be due to the reason that post-prandial blood glucose response is dose dependent (83). The composition of the quinoa-incorporated MG bread of the present study was refined wheat flour (52%), whole wheat flour (25%), and quinoa flour (20%). Farinazzi-Machado et al. (84) showed that long-term consumption (30 days) of quinoa cereal bars led to a significant (*p* ≤ 0.05) reduction in blood glucose as quinoa bar was made with only quinoa and no other cereals.

Haemoglobin A1c levels between 5.7 and 6.4 % indicate pre-diabetes and a higher chance of getting diabetes. A person with levels of 6.5 % or higher indicates a person has diabetes (85). In the present case, subjects having HbA1c within the normal range both before and after supplementation were 5.46 ± 0.541 and 5.01 ± 0.677 mg/dL, respectively. Foods containing dietary fiber are associated with a reduction in the risk of diseases and can prevent hyperlipidemias, cardiovascular diseases, diabetes, and obesity (86–88). Carbohydrates from quinoa, including insoluble and soluble fiber, can be considered as nutraceuticals because they help in the reduction of blood glucose, triglyceride, total cholesterol, and free fatty acid levels in the blood (89). The results obtained in the present study are similar to the data reported in the literature, indicating that quinoa bread can be used to lower plasma lipids and glycemic control. Quinoa contains considerably high amounts of vitamin E, iron, zinc, and magnesium (90). These nutrients have shown hypocholesterolemic effects and increased postprandial sensitivity and release of plasma insulin (91–93). The presence of antioxidant capacity compounds, such as polyphenols, phytosterols, and flavonoids in grains of quinoa (94), might be the cause for the positive effects on reduction in plasma lipids such as total cholesterol, triglycerides, LDL and VLDL, and blood glucose levels in the subjects tested for biochemical parameters after the post-treatment with multigrain bread. Similarly, Farinazzi-Machado et al., (84) showed that after 30 days of treatment with a quinoa cereal bar, a significant reduction in blood glucose, cholesterol, triglycerides, and LDL and increased levels of HDL were observed. Increased consumption of phenolic compounds has been associated with a reduced risk of cardiovascular diseases and certain cancers (95, 96).

## 5 Conclusion

Based on the data obtained in this study, it can be concluded that the incorporation of quinoa into wheat bread results in essential health benefits. The incorporation level in the present study was dependent on the workability of the bread as well as acceptability, keeping the nutrient profile in mind. The increase in the percentage of incorporation of non-wheat flour decreased its acceptability. Of 14 formulations, TQ13 with an incorporation of 20% quinoa flour as well as 3% wheat bran was found to be satisfactory from both sensory and nutritional points of view. The GI of quinoa bread fell under the category of low-GI foods. Therefore, the maximum possible incorporation of quinoa in wheat-based bread helped in maintaining blood glucose levels with a non-significant reduction. Blood lipid profile of the individuals, especially LDL and VLDL, reduced significantly. There was no change observed for the values of HDL. These benefits have proved to be crucial in the dietary treatment of diabetes mellitus by resulting in improved glycaemic control as well as several metabolic parameters, such as improved blood lipid levels. High consumption of phenolic compounds has long been associated with reduced risk of cardiovascular diseases and certain cancers. Current trends in the enhancement of the antioxidant capacity of wheat bread by the addition of quinoa flour rich in phenolic compounds might play a beneficial role in the health status of a population. The results obtained in the present study corroborate the data reported in the literature, indicating that quinoa bread can be used in plasma lipids and glycemic control.

## Data availability statement

The original contributions presented in the study are included in the article/supplementary material, further inquiries can be directed to the corresponding author.

## Ethics statement

The studies involving humans were approved by Assam Agricultural University Ethical Committee. The studies were conducted in accordance with the local legislation and institutional requirements. The participants provided their written informed consent to participate in this study. Written informed consent was obtained from the individual(s) for the publication of any potentially identifiable images or data included in this article.

## Author contributions

NM: Conceptualization, Investigation, Writing – original draft. PD: Supervision, Writing – review & editing. MD: Writing – review & editing. LB: Writing – review & editing.
